# Development of a novel multiphysical approach for the characterization of mechanical properties of musculotendinous tissues

**DOI:** 10.1038/s41598-019-44053-1

**Published:** 2019-05-22

**Authors:** Malek Kammoun, Redouane Ternifi, Vincent Dupres, Philippe Pouletaut, Sandra Même, William Même, Frederic Szeremeta, Jessem Landoulsi, Jean-Marc Constans, Frank Lafont, Malayannan Subramaniam, John R. Hawse, Sabine F. Bensamoun

**Affiliations:** 1 Alliance Sorbonne Universités, Université de Technologie de Compiègne, Biomechanics and Bioengineering Laboratory, UMR CNRS 7338, Compiègne, France; 2 Université Lille, CNRS, Inserm, CHU Lille, Institut Pasteur de Lille, U1019 - UMR 8204 - CIIL - Center for Infection and Immunity of Lille, F-59000, Lille, France; 3 Centre de Biophysique Moléculaire, CNRS UPR4301, Orléans, France; 4 Sorbonne Université, Laboratoire de Réactivité de Surface UMR 7197, Paris, France; 5 Institut Faire Faces, EA Chimère 7516 UPJV, CHU Amiens Imagerie Médicale, Amiens, France; 6 Department of Biochemistry and Molecular Biology, Mayo Clinic, Rochester, MN USA

**Keywords:** Musculoskeletal models, Biological physics

## Abstract

At present, there is a lack of well-validated protocols that allow for the analysis of the mechanical properties of muscle and tendon tissues. Further, there are no reports regarding characterization of mouse skeletal muscle and tendon mechanical properties *in vivo* using elastography thereby limiting the ability to monitor changes in these tissues during disease progression or response to therapy. Therefore, we sought to develop novel protocols for the characterization of mechanical properties in musculotendinous tissues using atomic force microscopy (AFM) and ultrasound elastography. Given that TIEG1 knockout (KO) mice exhibit well characterized defects in the mechanical properties of skeletal muscle and tendon tissue, we have chosen to use this model system in the present study. Using TIEG1 knockout and wild-type mice, we have devised an AFM protocol that does not rely on the use of glue or chemical agents for muscle and tendon fiber immobilization during acquisition of transversal cartographies of elasticity and topography. Additionally, since AFM cannot be employed on live animals, we have also developed an ultrasound elastography protocol using a new linear transducer, SLH20-6 (resolution: 38 µm, footprint: 2.38 cm), to characterize the musculotendinous system *in vivo*. This protocol allows for the identification of changes in muscle and tendon elasticities. Such innovative technological approaches have no equivalent to date, promise to accelerate our understanding of musculotendinous mechanical properties and have numerous research and clinical applications.

## Introduction

Musculotendinous tissue is complex and consists of well-organized hierarchical structures that influence its function and mechanical properties. At present, the mechanical properties of muscle tissue are characterized *in vitro* using stretching tests (at different velocities) or contractile tests (using calcium) to measure the passive and active properties of the muscle^1–3^. However, few studies have performed transversal tests, such as indentation, to analyze the transversal elasticity of the tissue^4,5^. Atomic Force Microscopy (AFM) is a perfectly suited technique for carrying out such tests. Indeed, in the last two decades, AFM has emerged as a ubiquitous technique utilized in biological research under near-physiological conditions from single molecules to living cells^6–10^. In addition to its outstanding imaging capabilities, force-distance curve based modes are widely used to probe the mechanical properties of biological samples^11–13^. Using AFM, the viscoelastic behavior of skeletal muscle cells (myoblasts)^14^ and dystrophic muscle^15^ have been characterized using the Young’s modulus to quantify the functional benefit after gene therapy. At the level of muscle fibers, AFM has been used on fixed tissue samples from Mdx, or Col6a1 KO mice to analyze the mechanical properties of the sarcolemma^16^. However, it is difficult to compare the elastic values measured with AFM to other studies due to the experimental conditions such as preparation and fixation of the samples, type of cantilever employed and mathematical models used to extract the Young’s modulus from the force-distance curves which are specific to each protocol^17^. For the first time, we have devised a novel AFM protocol with the ability to be performed on intact living muscle and tendon fibers that can be used for numerous research applications across the field. We have also applied the most recent AFM data acquisition modes, more specially the Quantitative Imaging mode (QImode), in this new protocol allowing for the generation of high resolution elasticity maps. This technique highlights the distribution of the mechanical properties along the fibers and reveals the striated structure of the tissue.

However, AFM has limitations in that it is unsuitable for application on live animals. Thus, non-invasive imaging techniques such as elastography (magnetic resonance imaging (MRI) or ultrasound (US)) have begun to be used for *in vivo* estimation of the mechanical properties of mouse or rat muscle^18,19^. Elastography is based on the propagation of shear waves using an external driver placed on the muscle. Previous studies have implemented needle drivers implanted in the hindlimb of mice to acquire magnetic resonance elastography (MRE) based acquisitions for assessment of the mechanical anisotropic ratio of the shear storage moduli^18^. Recently, Nelissen *et al*.^20^ have developed a MRE-based approach to analyze skeletal muscle damage in rats. With regard to US elastography, a recent study^19^ has used the Aixplorer machine to show correlations between elastic properties and pathological characteristics of spinal cord injuries in rats. For ultrasound elastography, the driver is a probe placed on the tissue which generates localized acoustic radiation force to produce waves that propagate transversally and provide a two-dimensional real-time quantitative map of the elasticity of muscle. In the present report, we have utilized these technologies to devise a non-invasive US elastography protocol that allows for the characterization of muscle and tendon tissues mechanical properties in live animals. Ultrasound elastography is a more practical and available technique compared to MRE which requires the use of a MRI machine. Additionally, we have developed this protocol using a novel clinical probe that could allow for the use of this technique for clinical applications.

Typically, elastography and AFM protocols for assessment of skeletal muscle and tendon tissues are developed independently and therefore have substantial differences between them regarding machine settings, data analysis protocols etc. One goal of the present report was to develop a protocol that was identical for analysis of both skeletal muscle and tendon and that could therefore be universally applied to both tissues for mice.

In addition, we have also validated and correlated the elastic results with other analyses including MRI texture analysis (MRI-TA) and Transmission Electron Microscopy (TEM). For all of our studies, we have utilized wild-type (WT) and TIEG1 KO mice as our model system given their well-characterized biological and structural defects throughout the musculotendinous system. Such innovative technological approaches have no equivalent to date and promise to accelerate our understanding of musculotendinous mechanical properties.

## Results

### Atomic force microscopy (AFM) analysis of the elastic properties of musculotendinous tissues

**Figure 1:**
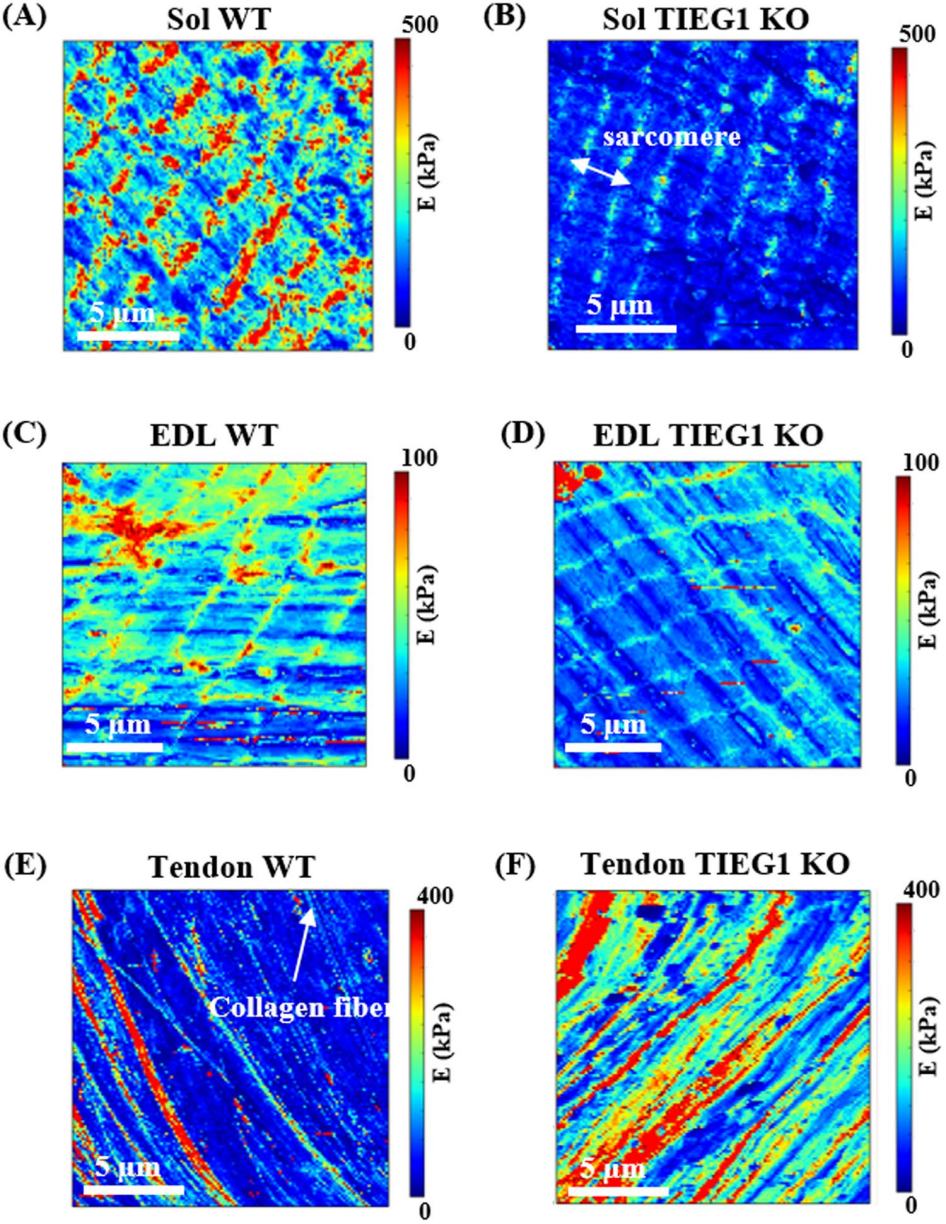
Elasticity maps (15 µm × 15 µm) obtained from the AFM protocol for slow (**A**,**B** soleus: Sol) and fast (**C**,**D** EDL) twitch muscle fibers and tendon fibers (**E**,**F**) of wild-type (WT) and TIEG1 knockout (KO) mice. The muscle and tendon cartographies are represented by different colors which are representative of lower (blue/green) and higher (red/orange) elasticities.

**Figure 2:**
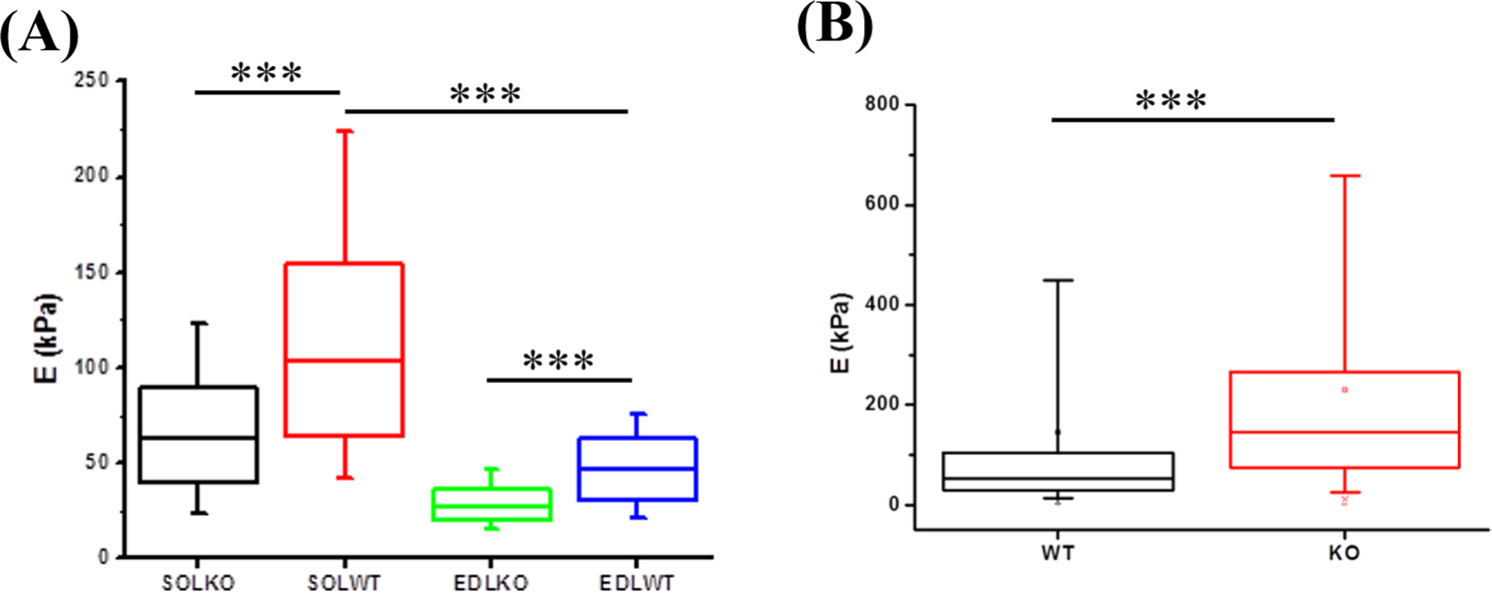
Quantification of Young’s modulus obtained via AFM for wild-type (WT) and TIEG1 knockout (KO) soleus (SOL) and EDL muscle fibers (**A**) and tendon (**B**) fibers. ***p < 0.001 between indicated groups.

AFM experiments were performed on skeletal muscle and tendon fibers to generate elasticity maps. The sarcomeric structure of muscle fibers was observed with this technique demonstrating its capability to measure elasticity of single muscle fibers (Fig. 1A–D). The Young’s modulus was significantly decreased for TIEG1 KO muscle fibers isolated from both the soleus and the EDL muscles compared to WT controls. This decrease in the Young’s modulus is represented by less intense color mapping for the TIEG1 KO fibers (Fig. 1). These analyses also revealed that slow twitch fibers isolated from WT soleus muscle have greater elasticity than fast twitch fibers isolated from WT EDL muscle (Fig. 2A). Conversely, TIEG1 KO tendon fibers exhibited a significant increase in the Young’s modulus compared to WT tendon fibers (Figs 1E,F and 2B). The Young’s modulus of the WT tendon (53.2 kPa) and WT EDL (46.5 kPa) were nearly identical indicating a similar range of the transversal elastic modulus for these two tissues. Finally, these results indicate that the impact of TIEG1 expression on elasticity is tissue specific.

### Transmission electron microscopy (TEM) analysis

**Figure 3:**
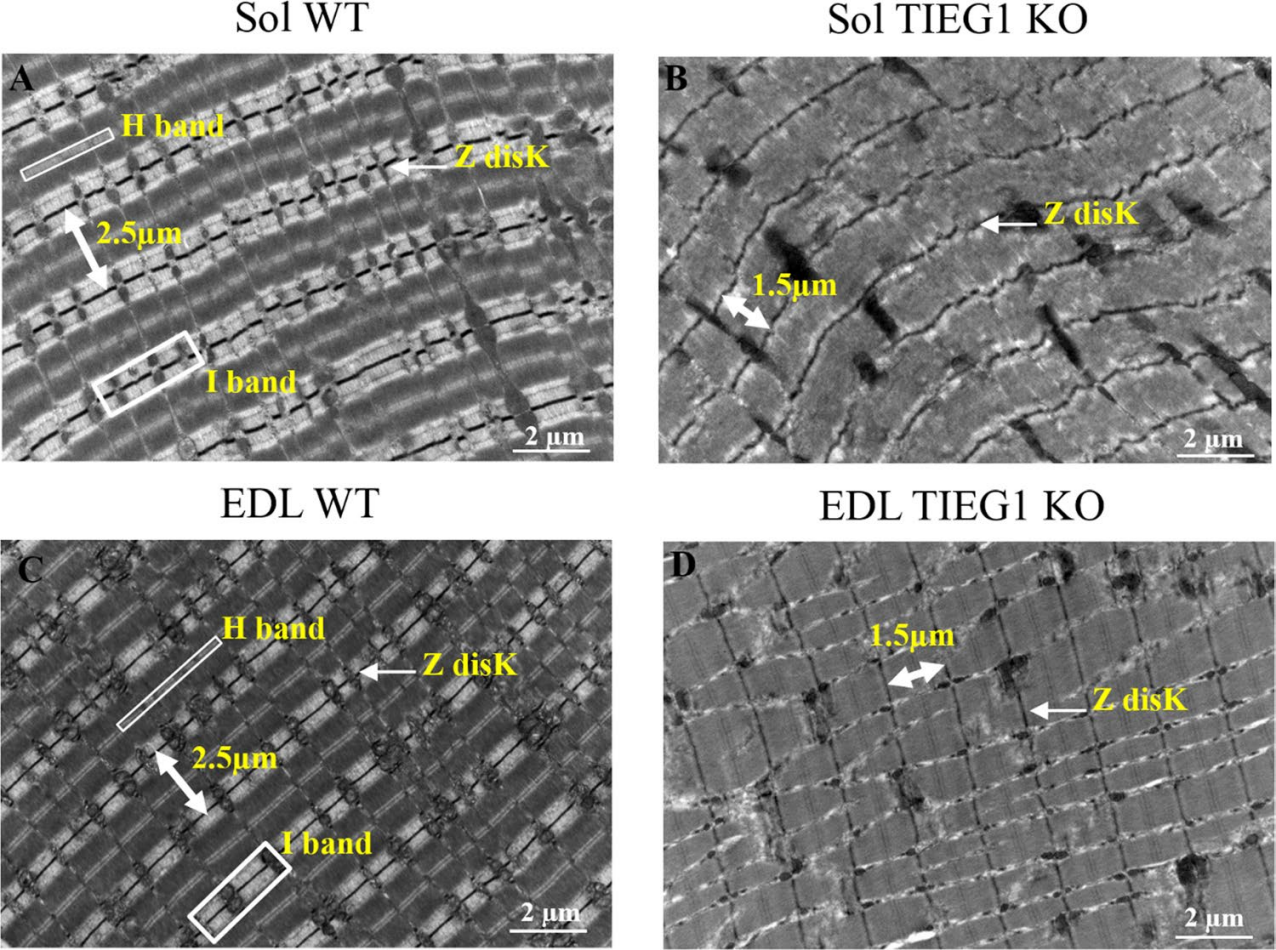
Transmission Electronic Microscopy (TEM) acquisition of soleus (Sol) and extensor digitorum longus (EDL) muscles isolated from wild-type (WT) and TIEG1 knockout (KO) mice. Longitudinal sections revealed a disorganized ultrastructure in both TIEG1 KO Sol and EDL muscles (**B**–**D**) compared to WT littermates (**A**–**C**).

In order to further understand the basis for the defects in the elastic properties of skeletal muscle tissues derived from TIEG1 KO mice via AFM, we performed TEM studies. Longitudinal sections of WT and TIEG1 KO soleus and EDL muscles revealed disorganization of the muscle structure in the absence of TIEG1 expression (Fig. 3). Specifically, smaller sarcomere lengths (1.5 µm for KO and 2.5 µm for WT) and the near complete disappearance of the I band were observed.

### Evaluation of the musculotendinous elastic and textural properties using ultrasound elastography

**Figure 4:**
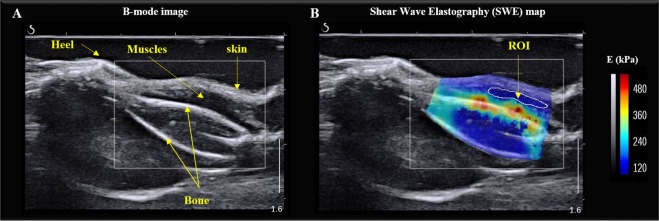
Representative B-mode image of the mouse hindlimb (**A**) and the corresponding elastogram (**B**) showing the elasticity of the different tissues present within the hindlimb. The region of interest (ROI) analyzed for quantification of the Young’s modulus (E) is indicated (**B**).

**Figure 5:**
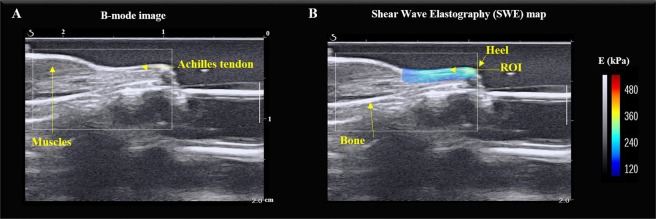
Representative B-mode image of the mouse hindlimb (**A**) and the corresponding elastogram (**B**) showing the elasticity of the different tissues present within the hindlimb. The region of interest (ROI) for measurement of the Young’s modulus (E) of the Achilles tendon is indicated (**B**).

**Figure 6:**
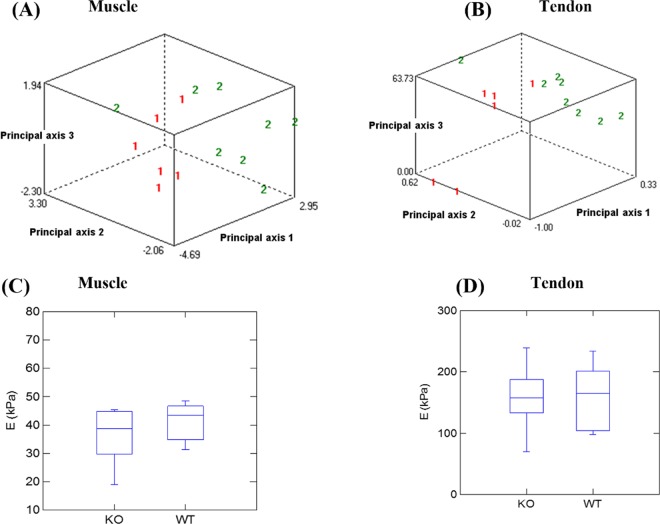
Principal component analysis of texture parameters from the B-mode acquisitions of muscle (**A**) and tendon (**B**) according to mouse genotype (1: TIEG1 knockout (KO) vs 2: wild-type (WT)). Significant differences for both tissues between the two genotypes are apparent. Quantification of muscle stiffness (**C**) and tendon stiffness (**D**) according to mouse genotype.

Given that AFM and TEM evaluation of tissues is performed on *ex vivo* tissues, we sought to evaluate the utility of ultrasound elastography of skeletal muscle and tendon tissues in live animals. From the B-mode image (Figs 4A and 5A), no significant difference of echo intensity mean or coefficient of variation were found between WT and TIEG1 KO tissues. Similar results were found for the contrast measured within the muscle region of interest (ROI) (TIEG1 KO: 54.5 ± 18.2% vs WT: 50.6 ± 14.1%) and the tendon ROI (TIEG1 KO: 40.9 ± 14.0% vs WT: 36.0 ± 9.7%). However, the TIEG1 KO and WT ROIs were well discriminated by texture analysis as highlighted in the PCA images (Fig. 6A,B).

From the elastography images, no significant difference in mean stiffness, coefficient of variation or contrast was observed. More specifically, no significant difference in the stiffness was detected between TIEG1 KO (36.1 ± 10.3 kPa) and WT (44.4 ± 13.4 kPa) muscle nor between TIEG1 KO (157.3 ± 56.3 kPa) and WT (161.1 ± 54.1 kPa) tendon (Fig. 6C,D). However, we noted significant stiffness differences between WT muscle (44.4 ± 13.4 kPa) and WT tendon (161.1 ± 54.1 kPa) with higher values observed in the tendon tissue.

### Hierarchical ascending classification (HAC) of skeletal muscles

**Figure 7:**
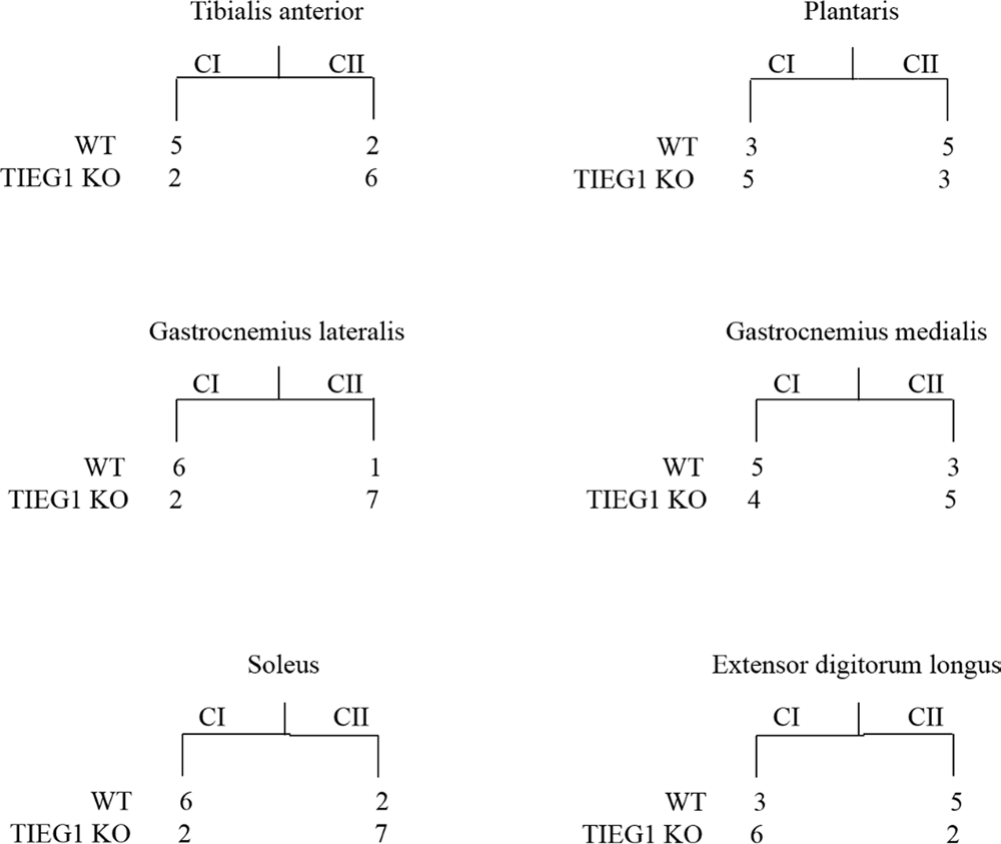
Hierarchical ascending classification (HAC) of the six muscles as a function of mouse genotype (wild-type (WT) vs TIEG1 knockout (KO)). Differences between Class I (CI) and Class II (CII) demonstrate a genotype effect on the different muscles.

Comparison of the Hierarchical Ascending Classifications (HAC) for all muscles (tibialis anterior, plantaris, gastrocnemius lateralis, gastrocnemius medialis, soleus and extensor digitorum longus) analyzed via MRI revealed two distinct groups of texture profiles which clustered as a function of mouse genotype (Fig. 7). Differences between Class I (CI) and Class II (CII) demonstrate a genotype effect on the different muscles. These results indicate different texture profiles between WT and TIEG1 KO muscles. The global values for the tibialis anterior, plantaris, gastrocnemius lateralis, gastrocnemius medialis, soleus, and EDL were found to be 73% (WT vs. TIEG1), 62.5% (WT vs. TIEG1), 81.2% (WT vs. TIEG1), 59% (WT vs. TIEG1), 76% (WT vs. TIEG1) and 70% (WT vs. TIEG1), respectively.

## Discussion

Muscle fibers are composed of different compartments (myofibril, extra sarcomeric cytoskeleton and sarcolemma) which ideally would be characterized in longitudinal and transverse directions in order to comprehensively characterize the entire fiber. However, with the use of existing technology and protocols, measurements in the transversal direction are often neglected despite the fact that important information could be obtained from such analyses for structural proteins located perpendicular to the fiber axis^21^. For instance, desminopathy is a muscle disease related to the defect of an intermediate filament (called desmin), which is aligned in a transverse manner through the Z-disks, known to play a critical role in the maintenance of structural integrity and mechanical properties^22^.

The AFM technique, which is primarily used to image biomolecular and cellular systems, is currently poorly utilized for analysis of mechanical properties in fibrous tissues. Past studies have employed AFM on tissue cryosections^4^ or following tissue fixation^5^ at resolutions of the whole tendon (diameter units: millimeter) or at the tendon fibril level (diameter units: nanometer). For the first time, we report the development of a novel protocol for the use of AFM on living tendon tissues at the resolution of single tendon fibers (diameter units: micrometer).

From an experimental standpoint, a challenge with such analyses is to immobilize the muscle fiber during the indentation test without using glue or chemical agents^16,23^ which could alter the mechanical properties of the tissue. The acquisition of the force curve during the indentation is also a key point of this technique. Indeed, the curve of the force, which is recorded for each pixel (128 × 128) of the cartography, must be validated according to the artifact of fiber movement leading to incorrect interpretation of the Young’s modulus. Another novel aspect of this study was to generate high resolution elasticity maps of single muscle and tendon fibers through the use of the QI mode of AFM. This allows for fast data acquisitions that are required to generate high resolution maps that highlight the spatial distribution of the musculotendinous mechanical properties.

The maps generated from these studies have revealed the regular periodic structure of the muscle fiber, with smaller sarcomeres (1.5 µm) observed in the TIEG1 KO muscle, and the collagenous parallel filaments within the tendon fibers. These structural observations are in agreement with TEM acquisitions performed on WT and TIEG1 KO muscle which revealed a disorganization of the muscle structure in both TIEG1 KO soleus and EDL muscles compared to WT littermates. Specifically, smaller sarcomere lengths (1.5 µm for KO and 2.5 µm for WT) and disappearance of the I band where observed in both slow and fast twitch TIEG1 KO skeletal muscles. These results demonstrate that the efficiency of the AFM protocols (experimental and acquisition) are representative of the main structures of muscle and tendon fibers with regard to the elastic cartography allowing for the avoidance of topographic acquisition that is currently performed with AFM.

Regardless of mouse genotype, slow skeletal muscle fibers (soleus) exhibited higher elasticity compared to fast fibers (EDL). These results most likely relate to the oxidative and glycolytic fiber composition of these specific skeletal muscles. In addition, the lower elasticity as detected by AFM in the TIEG1 knockout soleus and EDL fibers relative to the WT fibers may be reflective of the disorganized structure. The disorganization of TIEG1 KO skeletal muscle fibers likely leads to a decreased resistance under the tip of the AFM probe. The control (WT) values are of interest for numerical modelling, which need referent data to simulate the true mechanical behavior of biological samples. We also observed higher elasticity for the Z-disk region of the muscle structure compared to the M-band and this observation was in agreement with Ogneva’s study^21^ for rat muscle fibers.

In comparison with muscle fibers, the transversal elasticity of WT tendon fibers is within the same range as those of the WT muscle fibers. Interestingly, deletion of the TIEG1 gene increased the transversal elasticity of the tendon fibers compared to WT control fibers. Previous TEM studies on tendon tissue along the longitudinal axis of the tendon fiber did not reveal any structural changes between the dark and light bands^24^. However, Gumez *et al*.^25^ reported different X-ray diffraction patterns between WT and TIEG1 KO tendon fibers demonstrating that lack of TIEG1 expression results in a disorganization of the collagenous structure and a significant distortion of the triple helix network^26^.

It is interesting to note that deletion of TIEG1 has opposite effects on the elasticity of muscle vs. tendon fibers. Given the intimate association of these two tissues with one another, it is possible that these differences are due to compensation of one tissue for another. However, it is also possible that these effects are due to tissue specific differences exerted by this transcription factor. TIEG1 is known to mediate the down-stream effects of many cytokines and hormones including TGFβ, BMP, EGF, and estrogen among others^27–30^. It is therefore possible that loss of TIEG1 expression differentially impacts these signaling pathways in these two tissues. It is also possible that other transcriptional co-regulators and/or transcription factors that are essential for muscle and tendon development/function are differentially impacted by loss of TIEG1 expression. Additional studies are needed to identify the precise mechanisms by which loss of TIEG1 expression results in opposite effects with regard to the elasticity of muscle and tendon fibers.

To overcome the necessity to sacrifice mice, recent elastography imaging techniques (MRI, US) have enabled the measurement of mechanical properties in live animals. While magnetic resonance elastography has been used on muscles of mdx (Duchene) mice^18^, US elastography has never been performed on mouse muscle or tendon. In the present study, US elastography was performed with the Aixplorer machine which is clinically used for human tissues including muscle^31^ and sciatic nerve^32^ among others. Here, we have applied this technique to characterize the elastic properties of the musculotendinous system in a non-invasive manner. We have utilized a high frequency probe (SLH20-6) that has been recently adopted in clinical practice to image the functionality of superficial musculotendinous tissues. It should be noted that ultrasound imaging of mouse tissue is usually performed with homemade constructor probes. Using the SLH20-6 probe, muscle fiber structure and Achilles tendon were accurately analyzed with the use of the B-mode image (resolution 38 µm). Moreover, texture analysis revealed significant differences as a function of mouse genotype for both tissues, demonstrating that US elastography (Aixplorer) has the capability of identifying structural defects in muscle and tendon tissues. We have validated these findings with MRI texture analysis (MRI-TA) which also indicated significant structural changes between TIEG1 KO and WT muscle fibers. In comparison with the US technique, MRI has the ability to analyze the structure of all individual muscles within the hindlimb, while US can only characterize groups of muscles^33–35^. Interestingly, the hierarchical ascending classification (HAC) revealed that deletion of TIEG1 affected all muscle-texture profiles regardless of muscle type (oxidative: soleus, glycolytic: EDL, mix: gastrocnemius, plantaris, tibialis).

While US was able to detect structural changes as a function of genotype, the elastography acquisitions were not able to differentiate between WT and TIEG1 KO mice with regard to the elastic properties. This result indicates that US elastography, based on the analysis of shear waves, is not sufficiently sensitive to detect differences in elasticity. The development of higher frequency probes could allow for better spatial resolution and may allow for better resolution for detection of differences in elastic properties. Therefore, US elastography performed on the musculotendinous system cannot currently replace *in vitro* tests. However, this technique was able to differentiate the elasticity between tendon and muscle tissues regardless of mouse genotype. This differentiation was most likely related to the increased stiffness of the Achilles tendon compared to skeletal muscle.

It should be noted that the protocols developed in the present study have some limitations. Concerning AFM experiments, it remains difficult to compare the elastic modulus values with the ones obtained from similar experiments. This is primarily due to the numerous experimental conditions (type of AFM tip, calibration method, maximal indentation force, acquisition mode, mathematical models used to extract the Young’s modulus from the force-distance curves, etc …) which influence the measurement of the elastic properties. Nevertheless, this problem is being increasingly recognized, and some improvements have recently been proposed with the introduction of the Standardized Nanomechanical AFM Procedure (SNAP)^17^. We are perfectly aware of these limitations and for this reason we apply a series of precautions to ensure that our results are comparable from one experiment to another. This implies keeping the same parameters for the whole study, not changing the laser position and using the same type of cantilever (and where possible, the same cantilever) and the same mathematical model for the analysis.

As an extension of the results presented here, future studies will focus on developing the AFM protocol for analysis of muscle fibers under active conditions by altering calcium concentrations during the mechanical test. Furthermore, it is of interest to use this technique on tissues undergoing various degrees of stretching to provide a more dynamic measure of their functional properties. As for the US elastography protocol, the clinical probe utilized in this study did not allow for differentiating the elastic properties as a function of mouse genotype. To improve upon this limitation, it will be necessary to design a specific US probe with higher frequency that is compatible with the Aixplorer machine allowing for a better characterization of the mechanical properties of rodent soft tissues (muscle and tendon). In the future, it will be also of interest to adapt the present elastography protocols for other tissues.

In conclusion, we have developed a novel multiphysical approach that enables a better understanding of the structural and mechanical properties of soft living tissues including muscle and tendon. These novel applications will be useful in both research and clinical applications for the evaluation of normal and diseased states. Finally, through the use of these mouse models, we have further implicated important roles for TIEG1 in the musculotendinous system and have demonstrated that loss of TIEG1 expression reduces the elastic properties of muscle fibers while improving the transversal elastic properties of tendon fibers.

## Methods

### Mice and study design

The generation of TIEG1 KO mice has been previously described^36^ and initial characterization of TIEG1 expression and function in skeletal muscle and tendon were previously reported^24,25,37–41^. For the studies presented here, 3 month old littermate female animals from heterozygous breeding pairs were used. All mice were maintained in a temperature controlled room (22 ± 2 °C) with light/dark cycle of 12 hours. Animals had free access to water and were fed with standard laboratory chow ad libitum. This study was carried out in strict accordance with the recommendations in the Guide for the Care and Use of Laboratory Animals of the National Institutes of Health. The protocol was approved by the French ministry of higher education, research and innovation (Permit Number: DUO-4776) and the Mayo Clinic Institutional Animal Care and Use Committee (Permit Number: A9615).

### Atomic force microscopy (AFM) measurements

All experiments were performed on a commercial AFM (NanoWizard 3, JPK Instruments, Berlin, Germany) combined with an inverted optical microscope (AxioObserver.Z1, Zeiss) driven by JPK NanoWizard software 6.0 and Zen Blue 2012. Data were acquired in Quantitative Imaging mode (QI mode) using PFQNM-LC cantilevers with a nominal spring constant of 0.08 N/m and a 3.5 µm high conical tip with 8 nm tip radius (Bruker, Santa Barbara, CA, USA). Cantilever spring constant and sensitivity were calibrated before each experiment using the thermal fluctuation method of the JPK software, yielding spring constant values ranging from 0.019 to 0.025 N/m.

Control (N_WT_Tendon_ = 7, N_WT_____Soleus_ = 7, N_WT_____EDL_ = 7) and TIEG1 KO (N_KO_Tendon_ = 7, N_KO_Soleus_ = 7, N_KO_EDL_ = 7) fibers were extracted from slow (soleus) and fast (EDL) muscles^39^ as well as tail tendon^24^. Perfect immobilization of the sample is a prerequisite during each AFM experiment. This is particularly the case in experiments conducted on fibers where the slightest movement (vertical or lateral) is likely to generate artifacts during the acquisition of force curves, which will result in making the elasticity maps unusable. The absence of movement during transverse indentations is therefore a fundamental element of our experimental protocol. Fibers were immobilized on plexiglass and submersed in a drop of PBS. Bright field imaging was used to identify the area of interest on fibers. A visual control of the immobility of the fiber was performed using the optical microscope coupled with the AFM instrument. This ensured that only Quantitative Imaging (QI) files were recorded in a static condition. Subsequently, the AFM tip was engaged in the central portion of the fiber (to ensure a near plane contact surface to avoid curvature effects leading to artifacts) to scan an area of 15 µm × 15 µm. The AFM tip was scanned on three areas along each muscle and tendon fiber by applying a force trigger of 2 nN with a 3 µm Z ramp (i.e. the range between the two extreme positions of the AFM tip), at 80 µm/s. Compared to conventional Force-Volume (FV) mode, QI mode uses a specific movement algorithm that enables precise control of the vertical force at every pixel of the image without applying any lateral force that could damage soft biological samples. Combined with the new generation of short cantilevers, QI mode allows fast tip displacements, thus reducing the time required to capture elasticity maps. Hence, we were able to perform 128 × 128 pixel force mapping scans in less than 15 minutes while more than 5 hours would be needed for the same resolution with FV.

### Data analysis

The AFM data analysis was performed using in-house pyAF (python Atomic Force) software, version 1.5.1. An algorithm based on the fitting of the baseline was used to detect the point of contact on the force curve. A linear fit was made using the baseline of the approach curve. A noise threshold is used to shift the fit along the force axis. The noise parameter can be manually adjusted by the user to optimize the detection of the point of contact. The intersection between this shifted fit and the force curve defines the position of the point of contact. Subsequently, the elasticity was deduced from the Young’s modulus which was determined from the indentation portion of the curve using the elastic contact model for conical indenters (named Sneddon model^42^):

$$F=\frac{2}{\pi }\frac{E\,\tan \,\alpha }{1-{v}^{2}}{\delta }^{2}$$

where F is the measured force, E the elastic modulus, α the half opening angle of the tip, δ the indentation, and ν the sample’s Poisson’s ratio, that was set to 0.5.

### Elasticity maps

Arrays of force curves in the x and y planes (typically 128 curves × 128 curves) were recorded on areas of a given size (15 µm × 15 µm). From these force mapping scans, the Young’s modulus was then estimated for each force curve and displayed as colored pixels that reflect the magnitude of fiber stiffness (elasticity maps).

### Box plot

Box plots were generated by pooling all of the data used to create elasticity maps for each fiber type (tendon, muscle) (elastic modulus deduced from each individual force curve) as a function of mouse genotype. Unpaired t-test with Welch’s correction (which assume no equal SDs) was performed to compare elasticity between mouse genotypes for muscle (soleus, EDL) and tendon fibers (***p-value < 0.0001). Bottom and top of the boxes represent the first and third quartiles. The line within the box represents the second quartile, i.e. the median. The end of the whiskers represents the 9th and the 91st percentiles.

### Transmission electron microscopy (TEM)

Prior to processing for TEM, the soleus (N = 7) and the EDL (N = 7) muscles were dissected from WT and TIEG1 KO mice with both extremities pinned to avoid muscle contraction and to maintain original length. Muscles were then immediately placed in fixative [1% (vol/vol) glutaraldehyde and 4% (vol/vol) paraformaldehyde in 0.1 M phosphate buffer, pH 7.2]^43^ and incubated overnight at 4 °C. Subsequently, two pieces (1 mm × 2 mm) of muscle were dissected from the middle of the tissue and rinsed for 30 min in three changes of 0.1 M phosphate buffer, pH 7.2, followed by a 1 h secondary fixation in phosphate-buffered 1% OsO_4_ and 30 min in 1% uranyl acetate at room temperature. Following dehydration in a series of ethanol washes, tissue was embedded in EMbed 812 resin (EMS, Hatfield PA) and polymerized at 60 °C for 18h^44^. Ultrathin (90 nm) sections were cut using a Leica UC7 ultramicrotome (Buffalo Grove, IL) and placed on 200 mesh copper grids and stained with lead citrate. Five micrographs of each specimen were randomly captured across the muscle tissue using a JEOL 1400Plus TEM (Peabody, MA), operating at 80 kV with a magnification of 42000×. ImageJ 1.46/Java 8 software (National Institute of Health, Bethesda, MD, United States)^45^ was used to manually quantify the sarcomere length.

### Shear wave elastography imaging

**Figure 8:**
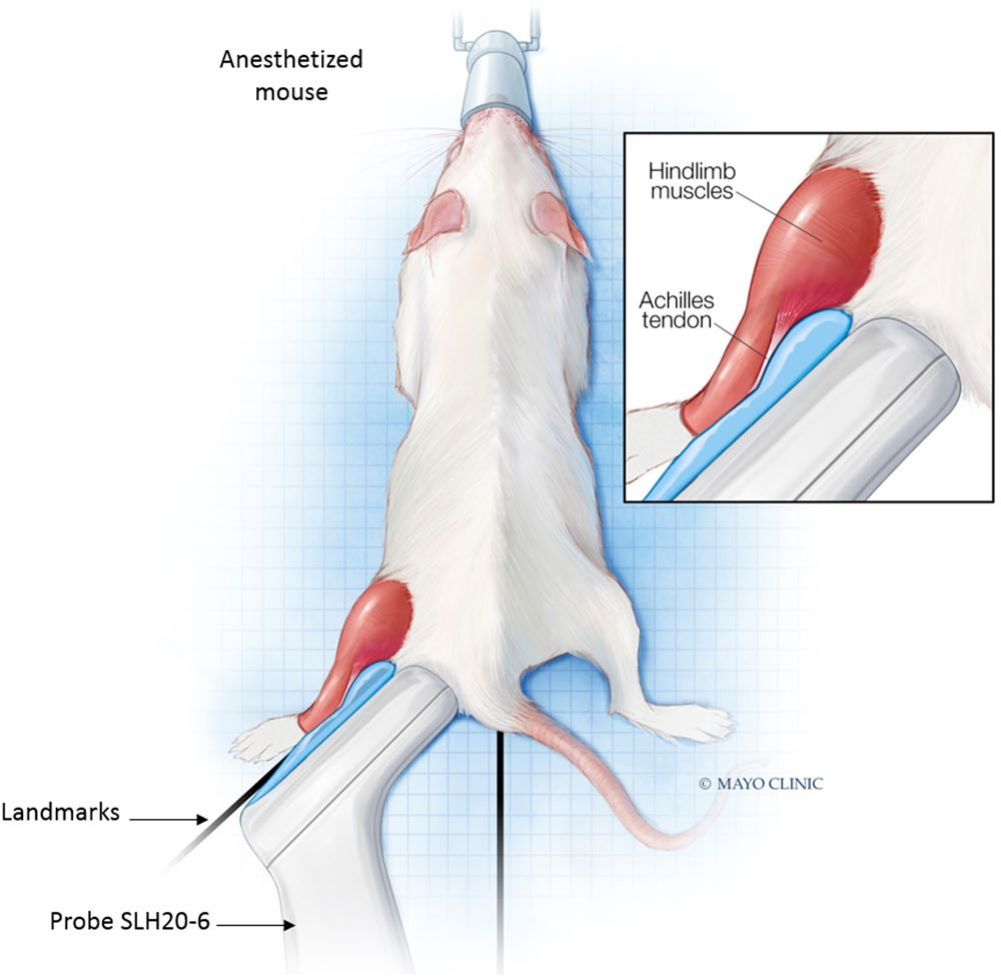
Model indicating the orientation and placement of the ultrasound probe on the mouse hindlimb. This image was drawn by Frank M. Corl from the department of Biomedical and Scientific Visualization, Mayo Clinic. Used with permission of Mayo Foundation for Medical Education and Research, all rights reserved.

Mice (N_WT_ = 7, N_KO_ = 7) were anesthetized with 1.5% isoflurane and a mixture of O_2_/air (1:1) at an output of 0.7 L/min and placed in a prone position. Landmarks were defined to orient the mice in a reproducible manner with the paw bent at a fixed angle (Fig. 8). Tendon and muscle in the right hindlimb were imaged by the same operator with an ultrasound machine (Aixplorer Multiwave^TM^ System, Supersonic Imagine, Aix-en-Provence, France) using the SLH 20-6 linear transducer probe having the specific characteristics: resolution: 38 µm, 2.38 cm footprint, 192 composite elements, effective bandwidth from 6 to 20 MHz.

For muscle imaging, the probe was placed on the surface of the hindlimb, which was shaved, parallel to the Achilles tendon. A thin layer of water-soluble transmission gel was applied for optimal acoustic coupling. To ensure that the probe was reproducibly placed, a visual control was performed using the B-mode image where the bone and the heel, considered as referent tissues, were identified. Subsequently, the same box size (10 mm × 10 mm) was defined for all acquisitions according to the length of the muscle and bone.

For tendon imaging, the probe was shifted to clearly visualize the Achilles tendon on the B-mode image. Bone and heel were also used as referent tissues and a box (10 mm × 10 mm) was placed in the same area for each mouse. The Aixplorer Multiwave^TM^ System generates two types of waves that propagate within the tissue: a compression wave that creates a high-quality B-mode image showing the anatomical structure within the hindlimb (skin, muscle, tendon, bone), and a shear wave that provides a quantitative color-coded map of tissue elasticity (Figs 7 and 8). The setting parameters for B-mode image were: ${\rm{pulse}}\,{\rm{duration}}=\frac{{\rm{number}}\_{\rm{half}}\_\mathrm{cycle}\,}{2\,Frequency}=600\,{\rm{\mu }}s$, gain 50%, pulse repetition frequency: 20 kHz and spatial resolution: 38 µm. To obtain mapping of elasticity with the shear wave elastography (SWE) sequence the setting parameters were: musculoskeletal preset, resolution mode enabled, tissue tuner at 1540 m/s, gain at 50%, dynamic range at 60 dB and the spatial resolution is 0.9 mm. The SWE mode is based on the generation of localized acoustic radiation force by the probe in the tissue; the locally excited tissue then produces waves which propagate transversally. In addition, the range of elasticity (Young’s modulus) was set between 0 kPa and 180 kPa, which corresponds to a shear wave velocity range of 0–7.7 m/s.

### Image analysis

In the B-mode image for US analysis (anatomical), a region of interest (ROI) was manually drawn within the hindlimb muscle group (lateralis and medialis gastrocnemius, soleus, EDL: Extensor Digitorum Longus, plantaris and tibialis) and within the surface Achilles tendon. ImageJ software (NIH, Bethesda, USA) was used to create the ROI. The ROI was then superimposed on B-mode images and elasticity images. Subsequently, the ROI Manager Tool of ImageJ was used to measure the following parameters: mean and standard deviation for echo intensity and stiffness, contrast (ratio of the difference (maximum - minimum) to the sum (maximum + minimum)) and coefficient of variation (ratio of standard deviation to mean).

The ROI of B-mode images were also analyzed for texture analysis using Mazda 4.6 software^45^, which includes several texture-analysis methods. In this study, gray-level histogram parameters and co-occurrence parameters were used. Principal Component Analyses (PCA) were performed to identify the most significant texture parameters that were able to discriminate KO muscle/tendon from WT muscle/tendon.

### Statistical analysis

The Systat^TM^ V11 software package (Systat Software Inc., CA, USA) was used to perform all statistical analyses. Non parametric two-sample Kolmogorov-Smirnov tests were performed in order to compare the elasticity values and the B-mode values between WT and TIEG1 KO mice. The statistical analysis was considered significant for P < 0.05.

### Magnetic resonance imaging (MRI)

#### Selection of skeletal muscles

MRI experiments were performed on 10 TIEG1 KO and 10 WT mice. For each mouse, muscles known to have different fiber types^46,47^ (slow, mixed, fast) were selected, namely tibialis anterior, plantaris, gastrocnemius lateralis, gastrocnemius medialis, soleus, and EDL muscles.

#### MRI acquisition

Prior to MRI analysis, mice were anesthetized with 1.5% isoflurane and a mixture of O_2_/air (1:1) at an output of 0.7 L/min. Respiration was monitored during the entire experiment, and body temperature was maintained at 37 °C using a warm-water circulation system. MRI was performed on a 9.4T horizontal ultra-shielded superconducting magnet dedicated to small-animal imaging (94/20 USR Bruker Biospec, Wissembourg, France) and was equipped with a 950 mT/m gradient set. A loop gap coil (10 mm inner diameter) was used for both proton transmission and reception. Axial images of the TIEG1 KO and WT hindlimb muscles were obtained using a gradient echo (Flash) sequence with the following parameters: TE/TR = 6 ms/252 ms, flip angle = 20°, FOV size = 2 × 2 cm, matrix size = 256 × 256, bandwidth = 50 kHz, slice thickness = 570 μm, to display 78 µm × 78 μm in plane resolution for a duration of 1 min (one accumulation).

#### Texture analyses

Acquired MR images were transferred to an external computer for data processing. ROIs were manually drawn as large as possible within the selected muscles and subsequently analyzed using Mazda software^48^ (Mazda 4.6, MRI analysis software, ©1998–2007 by P.M.Szczypinski), which includes several texture-analyses methods. In this study, the grey level histograms, composed of various parameters including mean, standard deviation, skewness, kurtosis and different percentiles were used. In addition, a co-occurrence matrix based on the following parameters: contrast, correlation, entropy, homogeneity, energy and run length matrix with run length and grey level non uniformity, long and short run emphasis, was applied.

#### Data analyses

PCA (principal component analyse) were performed to identify the most significant texture parameters able to discriminate TIEG1 KO muscles from the WT muscles with a confidence level of 0.95. A two-class (WT/TIEG1 KO) Hierarchical Ascending Classification (HAC) was performed with the most relevant texture parameters. This was performed for all muscles: tibialis anterior, plantaris, gastrocnemius lateralis, gastrocnemius medialis, soleus and extensor digitarum longus. The global value (true positive + true negative/total number of mice) was calculated for each HAC. This parameter represents the number of mice that are grouped into WT or TIEG1 KO by means of their texture profiles. A value superior to 60% is considered good.
